# Validation of a quantitative liquid chromatography/hybrid quadrupole‐linear ion trap MS/MS method for carfentanil and caffeine in a live human epidermis model

**DOI:** 10.1002/rcm.9420

**Published:** 2022-12-07

**Authors:** Jonathan M. Oyler, Kathleen J. Maistros, David Kilgour

**Affiliations:** ^1^ Nottingham Trent University Nottingham UK; ^2^ Laboratory Services Department U.S. Army Medical Research Institute of Chemical Defense Aberdeen MD USA

## Abstract

**Rationale:**

Carfentanil, an opioid 10K times more potent than morphine, has no licit clinical use. A powerful CNS depressant, it has been identified increasingly as the cause of overdose death in the United States. Because it is highly lipophilic, the law enforcement and medical communities have been concerned that responding personnel could be percutaneously exposed and that exposure could be enhanced with the use of alcohol‐containing hand sanitizers. An LC‐MS/MS method was developed to evaluate solvent effects on percutaneous absorption of carfentanil in a live human epidermal model.

**Methods:**

In this study, a quantitative liquid chromatography/hybrid triple quadrupole‐linear ion trap method was developed for carfentanil and for caffeine, a molecule routinely used to monitor epidermal cell culture viability. The method employed reverse‐phase liquid chromatography coupled with positive electrospray ionization and multiple reaction monitoring (MRM) to quantify carfentanil and caffeine against calibration curves formulated from authentic standards. Limits of detection (LOD) for the two compounds were determined using 10:1 signal‐to‐noise requirements for all product ions with relative peak areas within ±20% of those observed for a mid‐level calibrator. Precision and accuracy were determined by analyzing positive controls formulated in quintuplicate, by a different analyst, at three concentrations bracketing the method dynamic range. Inter‐day precision was evaluated using data collected from three separate days of analyses.

**Results:**

Calibration curves for seven formulated replicates of the two compounds met linearity requirements over at least four orders of magnitude concentration range. The accuracy of measured concentration results was within ±20% of the actual, precision across results (%CV) was ≤15%, curve coefficients of determination (*r*) were ≥0.980 (correlation coefficient *r* > 0.990), and relative ion ratios of all qualifier ions were within ±20% of those for a mid‐level standard. Limits of quantification (LOQs) for carfentanil and caffeine were 230 pg/ml and 12 ng/ml, respectively. Intra‐day accuracies (mean concentrations) for carfentanil and caffeine ranged from 90.1% to 100.8% and from 87.1% to 108.9%, respectively; inter‐day accuracies ranged from 98.7% to 100.4% and from 97.5% to 101.7%, respectively. Intra‐day precision (%CV) over the dynamic range ranged from 1.31 to 8.88 and from 1.49 to 6.72 for carfentanil and caffeine, respectively. Inter‐day precision ranged from 4.7 to 9.9 %CV for carfentanil and 7.6‐121 %CV for caffeine.

**Conclusions:**

The method was used to evaluate the percutaneous absorption kinetics of carfentanil in solution as a function of solvent composition using an in vitro, live human epidermis model. Counterintuitively, as previously reported, the addition of organic solvents to the formulations decreased rather than increased the percutaneous absorption rate of the ultra‐potent opioid, carfentanil.

## 1 INTRODUCTION

One of the major strengths of quadrupole mass spectrometry is its ability to rapidly and reproducibly scan through relatively wide mass‐to‐charge ranges with great sensitivity and speed, especially when targeting limited characteristic ions using a selected ion monitoring mode. The subsequent development of triple quadrupole instruments resulted in the ability to reproducibly fragment a precursor molecule under controlled conditions and quantitatively measure the resultant product ions to characterize and quantify the parent molecule with even higher specificity and sensitivity.^1^ Consequently, triple quadrupole instruments have become the “gold standard” for quantification of drugs and small molecules in toxicological, forensics, drug monitoring, and pharmaceutical analyses. In this study, a highly sensitive quantitative liquid chromatography/hybrid triple quadrupole‐linear ion trap MS/MS method was validated for carfentanil and caffeine. Subsequently, the method was used in an in vitro protocol designed to evaluate percutaneous absorption kinetics of carfentanil in a live human epidermal model, as a function of solvent composition.

Fentanyl analogs, like other opiates and opioids, act directly on central nervous system μ‐opioid receptors to induce bradycardia, respiratory depression, hypotension, depressed cough reflex, miosis, and sedation.^2^, ^3^, ^4^ An extremely potent fentanyl analog, carfentanil (methyl 4‐(1‐oxopropyl) phenylamino‐1‐(2‐phenylethyl)‐4‐piperidine carboxylate‐2‐hydroxy‐1,2,3‐propanetricarboxylate) is a DEA Schedule II opioid (synthetic drug) that has been estimated to be 100 times more potent than fentanyl and 10 000 times more potent than morphine, a naturally occurring opiate.^5^ The citrate salt was originally formulated (Wildnil) for use in veterinary medicine as a large animal tranquilizer but has not been commercially available since 2003. The intravenous LD_50_ of the citrate salt in mice and rats has been reported to be 3.39 and 18.75 mg/kg, respectively,^6^ and the subcutaneous median effective dose (ED_50_) required to induce severe intoxication in African green monkeys was found to be 0.706 μg/kg.^7^ Due to its ultra‐potency and because it has no licit clinical uses, the clinical LD_50_ of carfentanil has not been determined, but the estimated human lethal dose is 20 μg (0.286 μg/kg), with significant clinical effects observed with exposure to as little as 1 μg.^5^ This toxicity is within the range of classic organophosphate nerve agents, making it a potential agent of warfare and terrorism. There have been terrorist incidents in which humans have been exposed to carfentanil. In 2002, Chechen hostages in a theater in Moscow were presumably exposed to vaporized or aerosolized carfentanil and remifentanil, another highly potent opioid, resulting in 170 human deaths. Urine and clothing residue collected from three victims, analyzed by scientists at Porton Down, Wiltshire, England, contained the two fentanyl analogs.^8^, ^9^

Recently in the United States, the incidence of fentanyl‐related overdose deaths has dramatically increased.^10^ In the past this resulted from the exposure of individuals to fentanyl‐adulterated heroin, and cocaine and other psychostimulants (primarily methamphetamine),^11^ but increasingly, especially during specific localized outbreaks, fentanyl analogs alone, including carfentanil, have been implicated.^4^ With this dramatic increase in overdose cases, concern has grown for the safety of responding law enforcement and emergency medical personnel due to potential occupational exposure as these highly potent opioids are known to be readily absorbed through skin and mucous membranes. Incidents of percutaneous and dermal fentanyl exposure have been reported. There have been multiple reports from emergency response personnel describing symptoms consistent with opioid exposure presumably from percutaneous, dermal, and mucous membrane contact with on‐scene material and/or potentially contaminated surfaces.^12^, ^13^ In a confirmed case resulting from skin and mucous membrane exposure, a veterinarian's facial skin and mucous membranes of the eye and mouth were exposed to a large animal veterinary formulation that splashed from a misfired dart. The individual exhibited classic opioid symptoms, including nausea, respiratory depression, miosis, and hypotension.^14^

In addition, because fentanyl analogs are lipophilic and readily absorbed through the skin, concern has been generated in the medical community that the routine use of alcohol‐containing hand sanitizers by law enforcement and emergency medical personnel could enhance dermal absorption rates. For this reason, the NIOSH, NIH, the US DEA, and the NIEH have issued guidance documents strongly discouraging the use of alcohol‐based hand sanitizers by first‐responders who are at risk of being exposed occupationally to fentanyl‐related substances.^15^, ^16^, ^17^

Due to its potency, until recently no human data from controlled studies describing percutaneous or dermal absorption of carfentanil existed. For this reason, an in vitro epidermal dosing study was designed and conducted using a live, reconstructed human epidermal model (RhE) to measure percutaneous carfentanil permeability coefficients, absorption rates, and permeation lag times as a function of the solvent in which it was formulated.

**FIGURE 1:**
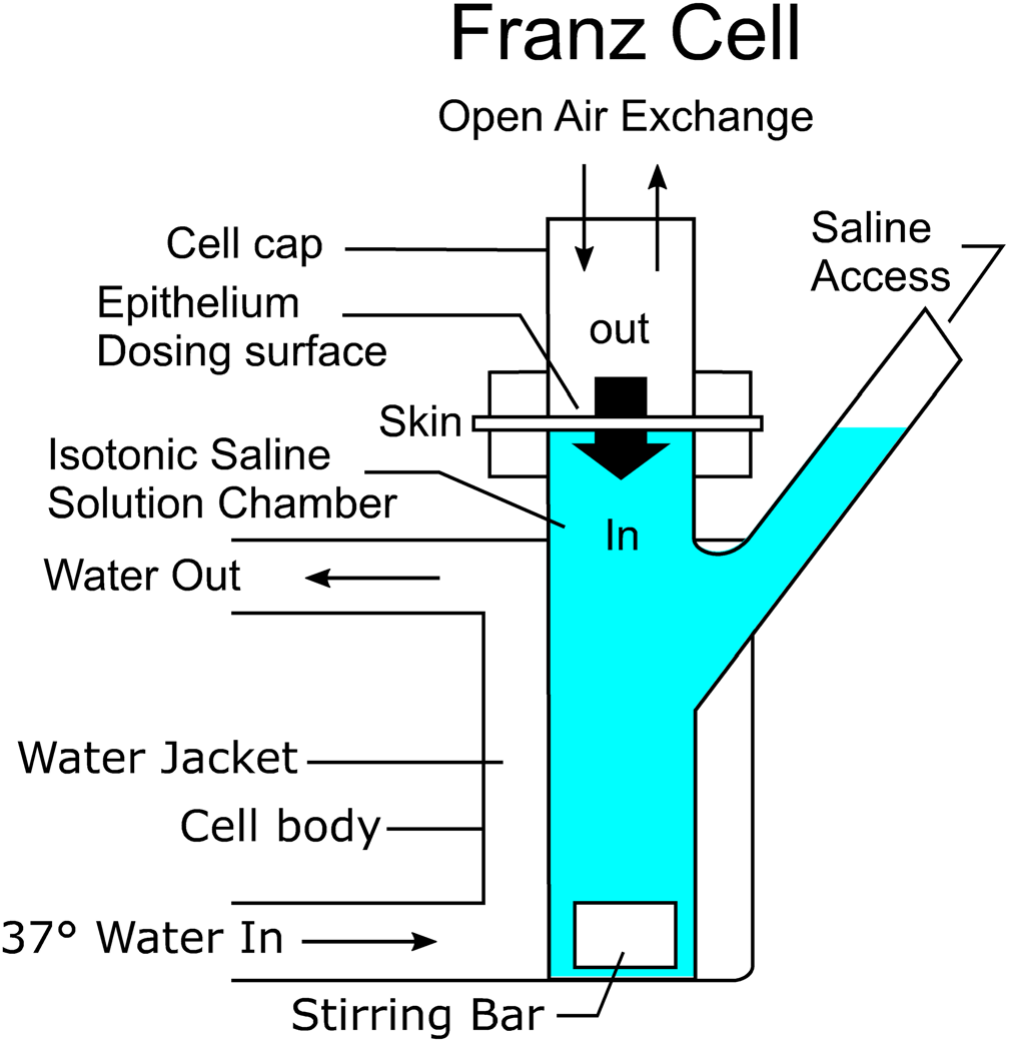
Franz Cell live human epidermal model [Color figure can be viewed at wileyonlinelibrary.com]

The in vitro Franz Cell system consisted of two chambers with an air/liquid interface between the upper and lower chambers (Figure 1). Live, cultured human epidermis with intact stratum corneum was suspended, stratum corneum side up (air side), at the air/liquid interface on Krebs‐Ringer bicarbonate solution where cell integrity and viability could be maintained; caffeine was employed as a cell viability marker.^18^ Dosing was performed in the upper chamber, onto the air‐exposed stratum corneum, and samples were drawn from the lower chamber through a port, prior to and at different time points after dosing. Epidermal cells were also dosed with caffeine, a drug routinely used to monitor epidermal viability in this model. This provided a live human model in which absorption kinetics could be studied as a function of dosing formulation solvent composition. In the in vitro study, carfentanil was formulated in four vehicles, water, ethanol, and two ethanol‐containing hand sanitizers, for dosing. The pharmacokinetic results have been reported.^19^

Prior to conducting the pharmacokinetic study, a sensitive LC/hybrid triple quadrupole‐linear ion trap (LC–MS/MS) method for direct quantification of carfentanil and caffeine in the RhE buffer was designed and validated. The method incorporated another fentanyl analog, sufentanil, as an internal standard (IS). (Theoretically, the use of an isotopically labeled carfentanil as an IS should produce comparable or better method performance.) This report describes the method validation process and method performance in detail.

## 2 MATERIALS AND METHODS

Carfentanil citrate (CAS no. 61380‐27‐6; purity: 96.9%) was obtained from Edgewood Chemical and Biological Center. Caffeine (CAS no. 58‐08‐2) was purchased from Sigma (St. Louis, MO, USA). Sufentanil citrate (CAS no. 60561‐17‐3) was purchased from Cerilliant Corporation (Round Rock, TX, USA) for use as the analytical IS. Water (18 MΩ) was produced by an in‐house Millipore system acquired from MilliporeSigma, Burlington, MA, USA. LC–MS‐grade methanol, acetonitrile, and formic acid were purchased from Fischer Scientific (Hampton, NH, USA). All test systems, reagents, and chemicals were maintained according to manufacturer's instructions.

## 3 STANDARDS

Carfentanil and caffeine primary stock solutions were gravimetrically formulated from neat stock in ethanol and stored at −20°C in borosilicate vials until further use. The IS, sufentanil, was commercially acquired in solution at 100 μg/ml in methanol (Lot FE013012‐01), in flame‐sealed borosilicate vials. Stock standards were formulated volumetrically using calibrated Eppendorf pipettes. Calibration standards and controls were diluted volumetrically in water containing 0.1% formic acid fresh each day of analysis from primary standards. Calibration curve standards for carfentanil and caffeine ranged in concentration from 0.23 to 920 and from 11.88 to 19 800 ng/ml, respectively.

## 4 ANALYTICAL METHOD

### 4.1 LC method

The LC system used was a Shimadzu Nexera UHPLC. A 5 μl sample aliquot was analyzed on a Phenomenex Kinetex column (2.1 × 100 mm × 1.7 μm F5, PN: 00D‐4,722‐AN, SN no. H18‐042762). Mobile phase A consisted of MS‐grade water with 0.1% formic acid, and mobile phase B consisted of MS‐grade acetonitrile containing 0.1% formic acid. The mobile phase flow rate was 0.4 ml/min using an initial 0.1‐min hold time at 2% mobile phase B, a gradient profile of 2%–90% mobile phase B over 4 min, and a 30°C column temperature.

### 4.2 MS/MS method development

**FIGURE 2:**
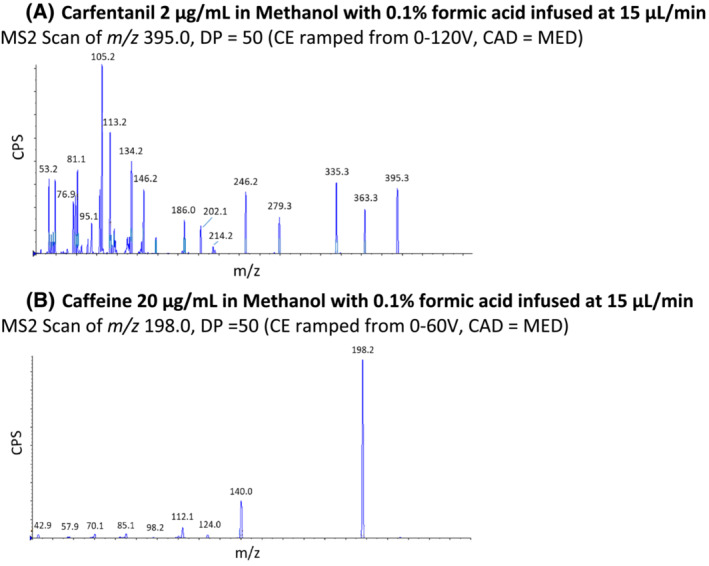
Summed product ion scan spectra of carfentanil and caffeine [Color figure can be viewed at wileyonlinelibrary.com]

**FIGURE 3:**
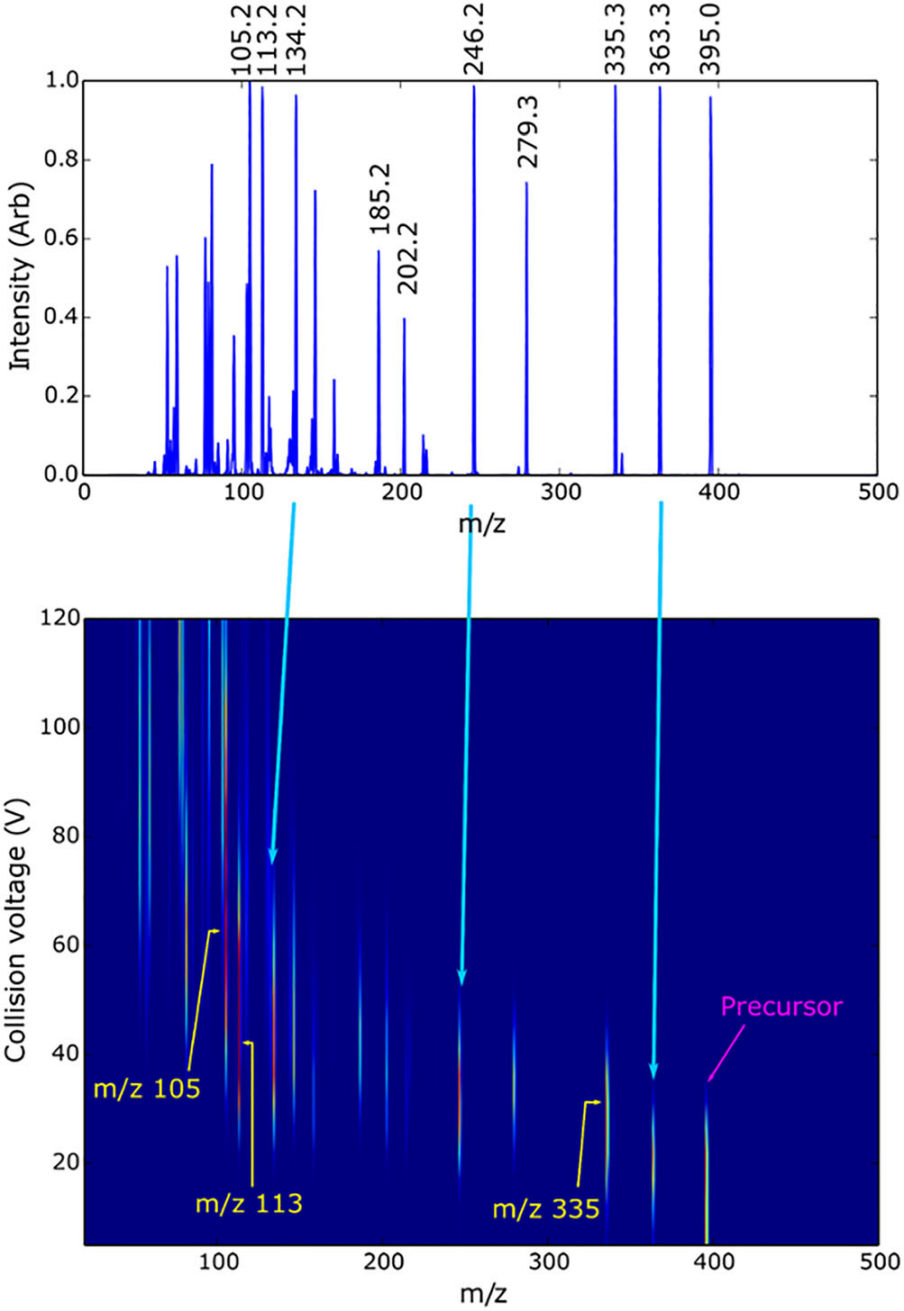
Carfentanil total fragment ion mass spectrum and heat map of product ion intensity as a function of CE [Color figure can be viewed at wileyonlinelibrary.com]

The MS/MS system employed for carfentanil and caffeine quantification was a SCIEX 4000 QTRAP, a nominal mass accuracy/resolution hybrid quadrupole‐linear ion trap, operated in positive ESI mode. To develop the method, standards at 2 μg/ml in methanol with 0.1%FA were directly infused at 15 μl/min and analyzed in Q1 full scan mode to acquire a background‐subtracted spectrum for each compound. The (M + H)^+^ species for each compound was identified to use as a precursor ion for MS/MS analysis; standards were directly infused to optimize source and Q1 conditions for each respective precursor. Again, samples were directly infused in product ion scan mode (precursors selected in Q1 followed by collision‐assisted dissociation [CAD] in Q2 with the resulting product ions scanned out of Q3 to identify the most abundant characteristic product ions for use in multiple reaction monitoring [MRM] analysis). During product ion scanning for both analytes, the declustering potential (DP) was held at 50, and the collision energy (CE) was ramped in 5 V steps from 0 to 120 V for carfentanil and from 0 to 60 V for caffeine. CAD of carfentanil produced many highly characteristic product ions to use for MRM analysis; again, CAD conditions were optimized for the selected ions. A heat plot of caffeine CAD produced a more limited number of prominent characteristic fragment ions. Figure 2 illustrates cumulative product ion scan spectra over the respective CE ranges for carfentanil and caffeine, and Figure 3 illustrates product ion intensities for carfentanil as a function of CE.

### 4.3 MRM method validation

MRM method quantification was performed with newly formulated calibration standards, quality control samples (QCs), and blank samples on a minimum of three separate days to measure inter‐day method performance; calibration standards and QC samples were formulated from stock solutions and analyzed each day. Calibration and QC stock solutions were formulated by different analysts. QCs consisted of low, medium, and high levels, bracketing the calibration curves, and were formulated by a different analyst. The IS, sufentanil, was spiked into the samples at a final concentration of 100 ng/ml prior to sample analysis.

A minimum of three characteristic product ions for each compound were selected for MRM. From the infusion experiments, characteristic product ions for both compounds were identified along with their corresponding optimal CEs. Subsequently, a high standard for each compound was analyzed using the LC gradient described earlier employing the identified optimal CEs. The most abundant characteristic fragment ion was selected as the quantitative transition; the second‐ and third‐most abundant characteristic fragment ions were selected as qualifier transitions for MRM spectral matching to ensure method specificity.

To determine the limit of detection (LOD) for carfentanil and caffeine, a minimum of seven daily replicates were analyzed. All blank controls had to contain no peaks interfering with the target analytes or ISs. At the method LOD concentration, a signal‐to‐noise criterion of ≥10:1 for quantifier and qualifier ions (sensitivity requirement) had to be met, and qualifier ion relative peak area ratios had to be within ±20% of those for a mid‐level calibration curve standard (spectral matching specificity requirement).

**FIGURE 4:**
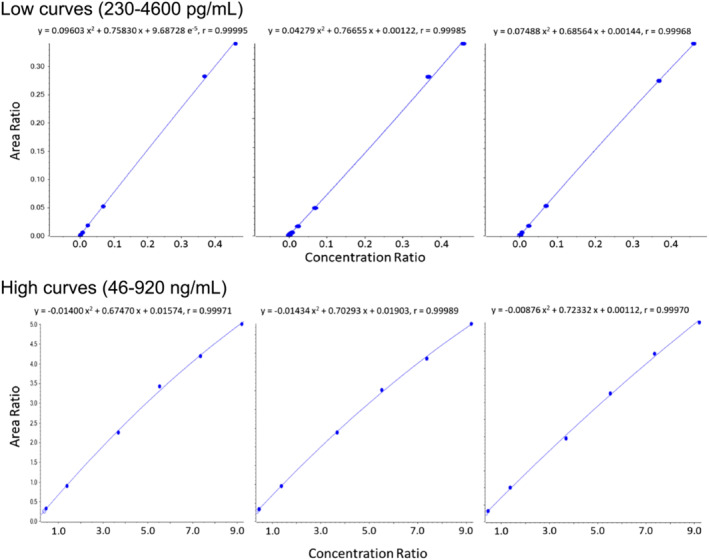
Daily carfentanil calibration curves [Color figure can be viewed at wileyonlinelibrary.com]

To identify the method limit of quantification (LOQ) and dynamic range, quantification criteria included requirements that all measured replicate results were accurate within ±20% of the actual, precision across results (%CV) was ≤15%, the curve coefficient of determination or r^2^ (measure of curve linearity) was ≥0.980 (correlation coefficient or r > 0.990), ion ratios of all qualifier ions were within ±20% of that for a mid‐level standard, and blanks contained no target analyte or IS signal above baseline. Two carfentanil calibration curves meeting all analytical criteria were employed to accurately quantify over the broad concentration range encountered with the study samples. Low and high calibration curve concentrations of carfentanil ranged from 230 to 4600 pg/ml and from 46 to 920 ng/ml, respectively, with each comprising a minimum of six calibration points. A single calibration curve meeting analytical criteria was used to quantify caffeine over a concentration ranging from 12 to 19 800 ng/ml. Figure 4 illustrates carfentanil calibration curves for the two dynamic concentration ranges employed in the 3‐day method validation. The curves were best described mathematically with quadratic formulae, using 1/x weighting for the area ratio term.

To determine method intra‐ and inter‐day precision and accuracy, a minimum of five replicate quality control samples at three concentrations bracketing the method dynamic concentration range were analyzed on three separate days. Intra‐ and inter‐day results were evaluated using LOD and LOQ requirements. Quality controls were formulated by a different analyst and evaluated against LOD and LOQ requirements. All calibrator and quality control results were required to meet the following accuracy and precision criteria: (a) all individual measured quantitative results had to quantify within ±20% of the expected concentration, (b) the mean measured concentration for each level had to quantify within ±15% of the expected concentration, and (c) the % coefficient of variation (CV) across replicate results had to be <15%.

## 5 RESULTS

Optimal source parameters for the target analytes included the following: curtain gas (CUR), 55 psi; ionspray voltage (IS), 5500 V; turbo gas temperature (TEM), 700 °C; gas one (GS1), 60 psi; gas 2 (GS2), 60 psi; interface heater (ihe), ON; CAD voltage, MED; entrance potential (EP), 10 V; and collision cell exit potential (CXP), 9 V. Table 1 lists the optimal MS/MS voltages for precursor and product ions.

**TABLE 1:** Optimal MS/MS parameters

Transitions	Precursor (M + H)^+^	Product ion	Dwell (ms)	Declustering potential (DP)	Collision energy (CE)
Carfentanil	395	335	62	60	30
		113			43
		105			63
Caffeine	195	138	62	60	15
		110			20
		83			41
Sufentanil	387	238	62	30	23
		355			23
		111			52

Applying both intra‐ and inter‐day linearity, accuracy, and precision acceptance criteria, low and high calibration curve concentrations of carfentanil ranged from 230 to 4600 pg/ml and from 46 to 920 ng/ml, respectively, with each comprising a minimum of six calibration points. A single calibration curve ranging from 11.88–19 800 ng/ml met analytical criteria and was used to quantify caffeine. All curves were evaluated as quadratic expressions with 1/x weighting, and correlation coefficients for all calibration curves exceeded 0.995. Intra‐day quantitative data listed in Tables 2, 3, 4 were evaluated using the curve regression equations; all calibration curve data met intra‐ and inter‐day precision and accuracy requirements.

**TABLE 2:** Carfentanil low calibration curve accuracy

Expected conc. (ng/ml)	Day 1		Day 2		Day 3	
	Mean conc. (ng/ml)	Accuracy (%)	Mean conc. (ng/ml)	Accuracy (%)	Mean Conc. ng/ml)	Accuracy (%)
0.046	**0.09**	**193.0**	**0.00**	**0.00**	0.042	91.25
0.069	**0.20**	**292.4**	0.068	98.53	0.065	94.62
0.23	0.21	91.8	0.24	104.25	0.27	116.43
0.69	0.74	107.5	0.68	98.76	0.72	104.80
2.30	2.34	101.9	2.31	100.25	2.17	94.35
6.90	6.83	98.9	6.72	97.44	6.73	97.51
36.8	36.73	99.8	37.55	102.04	37.77	102.63
46.0	46.06	100.1	45.42	98.74	45.27	98.41

*Notes:* A minimum of seven replicates/calibration level/day were analyzed to determine the limits of detection and limits of quantitation. Data in **red** did not meet accuracy requirements but did meet detection requirements. The valid carfentanil curve dynamic concentration range was 0.23–46 ng/ml.

**TABLE 3:** Carfentanil high calibration curve accuracy

Expected conc.(ng/ml)	Day 1		Day 2		Day 3	
	Mean conc.(ng/ml)	Accuracy(%)	Mean conc.(ng/ml)	Accuracy(%)	Mean Conc.(ng/ml)	Accuracy(%)
46	46.9	101.93	46.2	100.39	45.6	99.21
138	135.1	97.87	137.5	99.60	141.3	102.39
368	359.5	97.69	364.5	99.04	354.3	96.29
522	573.0	103.8	563.8	102.1	559.7	101.40
736	731.9	99.45	724.9	98.5	750.9	102.02
920	913.3	99.28	923.3	100.4	908.1	98.71

*Notes:* A minimum of seven replicates/calibration level/day were analyzed to determine the limits of detection and limits of quantitation. All data met accuracy requirements.

**TABLE 4:** Caffeine calibration curve accuracy

Expected conc.(ng/ml)	Day 1		Day 2		Day 3	
	Mean conc.(ng/ml)	Accuracy(%)	Mean conc.(ng/ml)	Accuracy(%)	Mean conc.(ng/ml)	Accuracy(%)
7.9	7.3	92.0	**3.3**	**41.9**	**11.4**	**143.5**
11.9	10.9	92.0	11.9	86.1	13.1	110.4
39.6	42.3	106.8	39.6	110.1	42.0	106.0
59.4	62.3	104.8	59.4	92.8	59.3	99.8
198	205.6	103.8	198.0	100.9	178.8	90.3
597	601	100.7	597.0	104.2	565.3	94.7
1584	1579	99.7	1584	105.7	1571	99.2
1980	2003	101.1	1980	102.5	1914	96.7
5940	5886	99.1	5940	97.5	6367	107.2
7920	7802	98.5	7920	101.8	7453	94.1
11 880	12 340	103.9	11 880	100.9	11 970	100.7
15 840	15 310	96.6	15 840	94.5	16 240	102.5
19 800	20 020	101.1	19 800	103.0	19 480	98.4

*Notes:* A minimum of seven replicates/calibration level/day were analyzed to determine the limits of detection and limits of quantitation. Data in **red** did not meet accuracy requirements in Table 7.

In addition, all daily QC sample results met precision and accuracy criteria during the validation. Intra‐day precision and accuracy data for carfentanil and caffeine QC samples from the 3 days of analysis are listed in Tables 5 and 6, and inter‐day precision and accuracy data for carfentanil and caffeine QC samples over the 3 days are listed in Table 7.

**TABLE 5:** Intra‐day carfentanil QC accuracy and precision

Expected conc.(ng/ml)	Day 1		Day 2		Day 3	
	Measured conc.(ng/ml)	Accuracy (%)	Measured conc.(ng/ml)	Accuracy (%)	Measured conc.(ng/ml)	Accuracy (%)
1.54	1.65	107.1	1.52	98.77	1.59	103.07
	1.51	98.3	1.51	98.20	1.66	108.00
	1.45	94.3	1.77	115.17	1.57	101.88
	1.48	96.1	1.44	93.44	1.42	92.24
	1.39	90.2	1.45	93.94	1.38	89.90
	**Mean accuracy = 97.20**		**Mean accuracy = 99.90**		**Mean accuracy = 99.02**	
	**%CV = 6.46**		**%CV = 8.88**		**%CV = 7.73**	
40.48	36.15	89.31	39.50	97.57	38.48	95.06
	36.27	89.60	39.68	98.03	39.92	98.63
	36.79	90.87	38.34	94.72	41.62	102.81
	36.04	89.03	40.41	99.82	38.59	95.32
	37.17	91.83	40.03	98.89	40.07	98.98
	**Mean accuracy = 90.13**		**Mean accuracy = 97.81**		**Mean accuracy = 98.16**	
	**%CV = 1.31**		**%CV = 1.97**		**%CV = 3.23**	
625.6	601.8	96.20	639.54	102.23	611.90	97.81
	597.1	95.45	609.06	97.36	595.30	95.15
	601.6	96.16	654.88	104.68	NA	NA
	622.6	99.53	615.65	98.41	597.80	95.55
	606.4	96.94	634.43	101.41	591.40	94.53
	**Mean accuracy = 96.86**		**Mean accuracy = 100.82**		**Mean accuracy = 95.76**	
	**%CV = 1.64**		**%CV = 6.46**		**%CV = 1.49**	

*Note*: CV, coefficient of variation; QC, quality control.

**TABLE 6:** Intra‐day caffeine QC accuracy and precision

Expected conc.(ng/ml)	Day 1		Day 2		Day 3	
	Measured conc.(ng/ml)	Accuracy (%)	Measured conc.(ng/ml)	Accuracy (%)	Measured conc.(ng/ml)	Accuracy (%)
132.78	117.4	88.38	152.4	114.79	126.6	95.31
	118.1	88.97	147.9	107.6	131.5	99.03
	116.7	87.92	156.7	118.03	129.3	97.39
	113.0	85.13	135.3	101.87	131.0	98.63
	113.2	87.23	135.7	102.2	123.0	92.62
	**Mean accuracy = 87.13**		**Mean accuracy = 108.9**		**Mean accuracy = 96.6**	
	**%CV = 2.08**		**%CV = 6.72**		**%CV = 2.75**	
1742	1522	87.37	1895	108.77	1615	92.71
	1563	89.72	1867	107.12	1655	94.99
	1536	88.16	1806	103.62	1751	100.49
	1542	88.51	1891	108.51	1638	94.00
	1623	93.17	1850	106.18	1766	101.36
	**Mean accuracy = 89.39**		**Mean accuracy = 106.84**		**Mean accuracy = 96.71**	
	**%CV = 2.55**		**%CV = 1.95**		**%CV = 4.08**	
13 464	12 660	94.02	13 711	101.84	14 950	111.02
	12 670	94.10	12 867	95.57	13 590	100.94
	12 720	94.47	13 891	103.17	NA	NA
	12 670	94.13	13 475	100.08	13 200	98.06
	12 260	91.08	13 375	99.34	13 780	102.34
	**Mean accuracy = 93.56**		**Mean accuracy = 100.00**		**Mean accuracy = 103.09**	
	**%CV = 1.49**		**%CV = 2.89**		**%CV = 5.41**	

*Note*: CV, coefficient of variation; QC, quality control.

**TABLE 7:** Inter‐day carfentanil and caffeine QC accuracy and precision

Analyte	Expected conc. (ng/ml)	Mean conc. (ng/ml)	Accuracy (%)	%CV
Carfentanil	1.54	1.52 ± 0.11	98.7	7.25
	40.48	38.74 ± 1.82	95.7	4.71
	625.60	628.10 ± 61.86	100.4	9.85
Caffeine	132.78	129.50 ± 13.50	97.5	10.40
	1742.0	1707.0 ± 130.0	98.0	7.63
	13 464.0	13 663.0 ± 12.1	101.5	12.12

*Note*: CV, coefficient of variation; QC, quality control.

## 6 DISCUSSION

A relatively sensitive and rapid quantitative LC/hybrid triple quadrupole‐linear ion trap MS/MS method was developed and validated for the extremely potent opioid, carfentanil, in a live human epidermal model previously designed and employed to perform pharmacokinetic measures after cutaneous drug exposure. The method was also validated for caffeine, which was, in turn, used to validate the epidermal model for EtOH‐containing solvents by verifying epidermal viability throughout the experiments. Another fentanyl analog, sufentanil, was employed as the IS for both target analytes. The method met rigorous sensitivity, specificity, accuracy, and reproducibility criteria required for quantitative methods used in toxicology, drug monitoring, pharmacokinetics, pharmacodynamics, and forensics. These data demonstrated the relative high sensitivity, accuracy, and reproducibility capabilities of triple quadrupole instruments for quantitative interrogation of samples for drugs and small molecules and illustrate why triple quadrupole instruments are considered the “gold standard” for quantification of small molecules in these disciplines.

The mass spectrometer used was a nominal mass accuracy/resolution hybrid quadrupole‐linear ion trap instrument. Not surprisingly, a single calibration curve proved to be too inaccurate at the lower and upper concentration ranges over the extended concentration range needed for carfentanil quantification. Therefore, “low” and “high” concentration curves were validated and employed. The curves, however, did each bracket at least four orders of magnitude concentration providing a very wide method dynamic range for the study. The caffeine calibration curve proved to be accurate and precise over the more than four orders of magnitude concentration range needed to verify epidermal cell viability during the exposures. Method accuracy and precision for carfentanil and caffeine met all acceptance criteria.

Counterintuitively, as reported previously,^19^ alcohol modifiers appeared to reduce permeation rates and peak concentrations in the RhE culture medium prolonging permeation lag times. The authors postulated that this was due to slowed partitioning from the hand sanitizers, due to reduced solvent polarity compared to water alone. The results appeared to indicate that the concern over increased risk for law enforcement and emergency personnel using these hand sanitizers in environments where they may be at risk from percutaneous carfentanil exposure may be unwarranted. These conclusions appeared to support previous results.^20^

### PEER REVIEW

The peer review history for this article is available at https://publons.com/publon/10.1002/rcm.9420.

## Data Availability

Data available on request from the authors The data that support the findings of this study are available from the corresponding author upon reasonable request.
